# WRKY Transcription Factors in *Jasminum sambac*: An Insight into the Regulation of Aroma Synthesis

**DOI:** 10.3390/biom13121679

**Published:** 2023-11-21

**Authors:** Zhaogeng Lu, Xinwen Wang, Salma Mostafa, Iqra Noor, Xinyi Lin, Shixiong Ren, Jiawen Cui, Biao Jin

**Affiliations:** 1 College of Horticulture and Landscape Architecture, Yangzhou University, Yangzhou 225009, China; zglu@yzu.edu.cn (Z.L.); mz120221434@stu.yzu.edu.cn (X.W.);; 2 College of Bioscience and Biotechnology, Yangzhou University, Yangzhou 225009, China

**Keywords:** aromatic compounds, jasmine, secondary metabolites, WRKY transcription factor

## Abstract

WRKY transcription factors are one of the largest families of transcription regulators that play essential roles in regulating the synthesis of secondary metabolites in plants. Jasmine (*Jasminum sambac*), renowned for its aromatic nature and fragrant blossoms, possesses a significant abundance of volatile terpene compounds. However, the role of the *WRKY* family in terpene synthesis in jasmine remains undetermined. In this study, 72 *WRKY* family genes of *J. sambac* were identified with their conserved WRKY domains and were categorized into three main groups based on their structural and phylogenetic characteristics. The extensive segmental duplications contributed to the expansion of the *WRKY* gene family. Expression profiles derived from the transcriptome data and qRT-PCR analysis showed that the majority of *JsWRKY* genes were significantly upregulated in fully bloomed flowers compared to buds. Furthermore, multiple correlation analyses revealed that the expression patterns of *JsWRKY*s (*JsWRKY27*/*33*/*45*/*51*/*55*/*57*) were correlated with both distinct terpene compounds (monoterpenes and sesquiterpenes). Notably, the majority of jasmine terpene synthase (*JsTPS*) genes related to terpene synthesis and containing W-box elements exhibited a significant correlation with *JsWRKY*s, particularly with *JsWRKY51*, displaying a strong positive correlation. A subcellular localization analysis showed that JsWRKY51 was localized in the nucleus. Moreover, transgenic tobacco leaves and jasmine calli experiments demonstrated that overexpression of *JsWRKY51* was a key factor in enhancing the accumulation of β-ocimene, which is an important aromatic terpene component. Collectively, our findings suggest the roles of *JsWRKY51* and other *JsWRKY*s in regulating the synthesis of aromatic compounds in *J. sambac*, providing a foundation for the potential utilization of *JsWRKY*s to facilitate the breeding of fragrant plant varieties with an improved aroma.

## 1. Introduction

Jasmine (*Jasminum sambac*) is an important ornamental plant of the Oleaceae family and well known for its ravishing fragrance [1]. Jasmine plants are economically valuable and are frequently utilized in the manufacturing of a variety of products, including perfumery, cosmetics, and food [2]. Moreover, jasmine tea is also a popular beverage in East Asia made from jasmine flowers [3,4]. It is widely cultivated for its scented flowers as shrubs and vines in tropical and subtropical regions [1]. Previous studies have mainly focused on analyzing the chemical composition of the jasmine floral scent, and approximately 100 chemical compounds have been identified. Among them, linalool, benzaldehyde, benzyl alcohol, benzyl acetate, nerolidol, citronellol, and α-farnesene are the most abundant in the extracts of *J. sambac* flowers [5]. However, further advancements in comprehending the genetic basis and molecular mechanism of *J. sambac* floral scent can be accelerated with the accessibility of genomic resources.

The floral fragrance is composed of volatile organic compounds or aroma compounds produced by floral tissues [6], whereas the terpenoids represent the largest group of volatile organic compounds in plants and account for a significant portion of the jasmine flower scent profile [7]. The terpenoids are produced in plants via two distinct pathways: the mevalonic acid (MVA) and 2-c-methylerythritol 4-phosphate (MEP) pathways. The MEP pathway produces monoterpene, carotenoid, and diterpene precursors in plastids, whereas the MVA pathway produces sesquiterpenes in the cytosol [8]. Several key structural genes involved in jasmine flower scent formation have been identified in earlier studies, especially within the terpenoid biosynthesis pathway. For example, some genes are responsible for the biosynthesis of α-farnesene (*JsHMGS*, *JsHMGR*, *JsFPPS*, and *JsTPS*) in the MVA pathway [9]. Similarly, *JsTPS3*, a terpenoid-related gene, has been found to enhance the β-ocimene production in jasmine [7]. In addition, transcription factors (TFs) such as MYB, bHLH, and WRKY families are also involved in the biosynthesis of terpenoids [10,11]. Recently, Qi et al., 2022 reported that many MYB-, bHLH-, and WRKY-binding elements were significantly enriched in promoter regions of *TPS* genes in single-petal *J. sambac* (cultivar, ‘Danbanmoli’) [11]. Collectively, TFs play an important role in the regulation of plant terpenoids by regulating the expression of functional genes.

WRKY TFs are one of the most abundant TF families in plants, interacting with numerous signaling networks to regulate plant growth and secondary metabolite synthesis [12]. The WRKY domain consists of approximately 60 amino acids that can bind the DNA through the W-box binding site with the core sequence TGAC to regulate the target genes [13]. The members of the WRKY family are mainly divided into three major distinct groups (I, II, and III) according to the number of WRKY domains and the type of zinc finger motifs. The proteins of group I contain two WRKY domains, whereas the members of groups II and III have only one WRKY domain [13,14]. The type of motif in groups I and II is the C_2_H_2_ zinc finger motif, while the C_2_HC zinc finger motif is in group III [15,16]. The WRKY TFs play important roles in regulating plant development, response to stresses, and secondary metabolite biosynthesis [12,17,18]. In this respect, many *WRKY* genes have been found to be involved in the regulation of terpenoid genes in several aromatic plant species [8,10]. For example, some monoterpenes in *Osmanthus fragrans* are regulated by OfWRKY TFs [16]. Similarly, CrWRKY1 positively regulates the biosynthesis of the terpenoid indole alkaloid in *Catharanthus roseus* [19]. Moreover, CoWRKY modulates the linalool synthesis in *Cinnamomum osmophloeum* [20]. In wine grapes, VviWRKY40 regulates monoterpenoid glycosylation [21]. Considering the importance of WRKY TFs in regulating terpenoid compounds and rich aromatic terpenes in *J. sambac*, an in-depth analysis of *WRKY*-related genes in sweet-scented jasmine needs to be explored.

Qi et al., 2022 [11] performed a genome-wide identification of *JsWRKY* genes in single-petal *J. sambac*, resulting in the identification of 69 *JsWRKY* genes. Another three potential WRKY genes related to stress were identified in the *J. sambac* genome (cultivar, Trifoliatum) [6]. Recently, a high-quality chromosome-level genome of *J. sambac* (cultivar, ‘double-petal’) was reported in our previous study [7], and 47 *TPS* genes containing two conserved domains were identified and characterized at the genome-wide level of *J. sambac*. Moreover, many aromatic compounds (terpene volatiles) and their abundance in flowering stages were also acquired in our previous study [7]. The available jasmine genomes and flower fragrances provide abundant resources for studying the regulation of terpene volatiles in jasmine species. To further identify the *WRKY* genes in double-petal *J. sambac* and characterize their roles in aromatic formation, we performed a genome-wide identification analysis of *WRKY* genes and then compared them between single-petal and double-petal phenotypes of *J. sambac*. We also performed multiple correlation analyses on the expression profiling of *JsTPS* and *JsWRKY* genes, as well as the expression of *JsWRKY* and terpene volatiles during the bud-to-full-blooming stages, to explore the involvement of the WRKY family in the formation of jasmine floral scent, specifically related to terpenoid compounds. Furthermore, the highly expressed *JsWRKY51* related to aroma synthesis was screened for subcellular localization and functionally characterized in tobacco plants and jasmine callus by genetic transformation. Our findings will provide potential genetic resources and facilitate further identification of crucial *WRKY* genes involved in the molecular mechanism of aroma production.

## 2. Materials and Methods

### 2.1. Identification of Putative WRKY Genes of J. sambac 

The hidden Markov model (HMM) file corresponding to the WRKY domain (PF03106) was downloaded from the Pfam protein family database (http://pfam.sanger.ac.uk/, accessed on 8 May 2023). HMMER 3.0 was used to search the *WRKY* genes from the *J. sambac* genome [7] with a cutoff E-value of 1 × 10^−5^. All candidate genes that may contain the WRKY domain based on HMMER results were further examined with CDD (http://www.ncbi.nlm.nih.gov/Structure/cdd/wrpsb.cgi, accessed on 10 May 2023) and Pfam (http://pfam.xfam.org/search#searchBatchBlock, accessed on 10 May 2023). The incorrectly predicted and redundant sequences were manually discarded. The basic physical and chemical parameters of the *J. sambac WRKY* genes were calculated with the online ProtParam tool. The reciprocal blast program (implemented in TBtools [22]) was used to search WRKY homologs between our study and that of Qi et al., 2022 [11], with the following cutoff criteria: weighted coverage > 0.5 and E-value ≤ 1 × 10^−10^.

### 2.2. Sequence Alignment, Phylogenetic Analysis, and Classification of JsWRKY Family

The protein sequences of the *Arabidopsis* WRKY proteins used for the phylogenetic analysis were downloaded from the Arabidopsis Information Resource (https://www.arabidopsis.org/, accessed on 10 May 2023). The AtWRKY and JsWRKY protein sequences were aligned using Clustal X2.1 with default parameters, and a phylogenetic tree was constructed using IQTREE (version 1.6.9) with the maximum likelihood method with a bootstrap value of 1000. All identified *JsWRKY* genes were divided into different groups according to the AtWRKY classification scheme.

### 2.3. Gene Structure and Motifs of JsWRKY Family

The exon–intron organization of *JsWRKY*s was analyzed using TBtools (version 1.098693) [22]. To identify conserved motifs in the JsWRKY proteins, the Multiple Em for Motif Elicitation (MEME) online program (http://meme.nbcr.net/meme/intro.html, accessed on 20 May 2023) was used for the protein sequence analysis. The optimized parameters were designed as follows: the number of repetitions, 0 or 1 per sequence; the motif width ranges from 10 to 60 amino acids; the maximum number of motifs, 10.

### 2.4. Genome Distribution and Gene Duplication of JsWRKY Family

All *JsWRKY* genes were mapped to *J. sambac* chromosomes based on physical location information from the jasmine genome database using the Circos plot. The gene duplication events were analyzed using the Multiple Collinearity Scan toolkit (MCScanX, version 1.0) with the default parameters. To exhibit the gene duplication and syntenic genes within the *J. sambac* genome, the duplication and synteny maps were constructed using TBtools. Non-synonymous (*Ka*) and synonymous (*Ks*) substitutions of each duplicated *WRKY* gene were calculated using KaKs_Calculator 2.0 [23].

### 2.5. Promoter Region Analysis of JsWRKY and JsTPS Family

Genome-wide identification of *JsTPS* genes in *J. sambac* was conducted in our previous study [7]. Based on the identified *JsTPS* genes, we further extracted their promoter sequences in this study. The promoter sequence, located 2000 bp upstream of the gene initiation codon, was selected from the *J. sambac* genome. Subsequently, cis-acting elements were analyzed using the Plant CARE online site (http://bioinformatics.psb.ugent.be/webtools/plantcare/html/, accessed on 25 June 2023). After data processing, the W-box element (the candidate WRKY binding site) was analyzed and then visualized using TBtools. In addition, the promoter sequence of *JsWRKY* genes was also analyzed using the same methods as mentioned above. 

### 2.6. Correlation Analysis between the Expressions of JsWRKYs and JsTPSs, as Well as between the Expression of JsWRKYs and the Abundance of Terpene Compounds 

To determine the expression changes of *JsWRKY*s during fragrance emission of *J. sambac*, the RNA-seq datasets of flower buds (FB, almost no fragrance emitted) and full-bloom flowers (FF, a large amount of fragrance emitted) were retrieved [7]. Based on the identified volatile terpenoids significantly accumulated in the FF stage [7], the correlation between the content of terpene compounds and the expression of *JsWRKY*s was estimated using Pearson correlation in this study. The potential regulatory network between them was constructed and visualized with Cytoscape (v3.9.1) [24]. Furthermore, the expression of *JsTPS*s at FB and FF was also retrieved, and their expression correlation with that of *JsWRKY*s was calculated using Pearson correlation.

### 2.7. Plant Materials and Growth Conditions

The *J. sambac* cultivar ‘double jasmine’, a major cultivated variety in China, was used in this study. The two-year-old jasmine plants were grown in a greenhouse under a 16 h light/8 h dark photoperiod and 25,000 lux of light intensity, at a 30/22 °C (light/dark) temperature and 50/80% relative humidity at Yangzhou University. Our previous study showed that almost no fragrance was emitted in the flowers at the bud stage (FB), but a large amount of fragrance was emitted from the semi-blooming stage (SF) to the full-blooming stage (FF) [7]. Therefore, the flowers at the SF and FF stages were selected as the main emission of fragrance in this study. The FBs (5–10 buds, stage I of Figure 1A in Chen et al., 2023 [7]), SFs (5–8 flowers, stage II of Figure 1A in Chen et al., 2023 [7]), and FFs (5–8 flowers, stage II of Figure 1A in Chen et al., 2023 [7]) were individually sampled from the same plant. Three healthy jasmine plants represent three biological replicates. The flowers at each stage from three replicates were used for qRT-PCR experiments. All samples intended for qRT-PCR experiments were immediately frozen in liquid nitrogen and then stored in a freezer at −80 °C. 

### 2.8. Quantitative Real-Time PCR Analysis 

To validate the expression changes of *JsWRKY*s during fragrance release of *J. sambac*, the total RNA of flower buds (FB), semi-bloom flowers (SF), and full-bloom flowers (FF) was isolated in accordance with the manufacturer’s instructions of the MiniBEST Plant RNA Extraction Kit (TaKaRa, Dalian, China). The cDNA was synthesized using the HiScript III RT SuperMix (Vazyme Biotech Co., Ltd., Nanjing, China) and was diluted with deionized water. *JsWRKY* gene primers were designed using Primer Premier 5.0 software (Table S7). qRT-PCR was performed as described previously [22]. The actin gene (*JsActin2* [25]) was used as an internal reference. All reactions were repeated for three biological replicates, and the comparative threshold cycle (Ct) was determined. Relative gene expression levels were determined using the 2^−ΔΔCt^ method.

### 2.9. Subcellular Localization Assay

For construction of the *35S::JsWRKY51*-*GFP*, the full-length coding sequences (CDSs) of *JsWRKY51* (without the stop codons) were amplified from the cDNA of *J. sambac* leaves. The vector construction was performed as described previously [26]. Briefly, the PCR products were first inserted into the intermediate vector pMD-19 T, and then the plasmid was digested using BamHI, and the fragments were inserted into pRI101-GFP to generate *35::JsWRKY51*-*GFP* via the Exnase II Cloning Kit (Vazyme Biotech, Nanjing, China). About 25-day-old tobacco (*Nicotiana benthamiana*) seedlings were selected for transformation. The *35S::JsWRKY51*-*GFP* and empty pRI101-GFP vectors (used as a control) were introduced into the fully grown upper leaves of tobacco using the *Agrobacterium* (GV3101)-mediated transformation method [27]. The GFP fluorescence signal was monitored under a confocal laser scanning microscope (LSM880; Carl Zeiss, Jena, Germany) 48 h after transformation. 

### 2.10. Gene Functional Characterization and Measurement of β-Ocimene Content with GC-MS

To validate the function of the *JsWRKY51*, the *35S::JsWRKY51*-GFP constructs and an empty vector (control) were introduced into the 25-day-old tobacco leaves using the *Agrobacterium* (GV3101)-mediated transformation method. Three days after transformation, the expression of the *JsWRKY51* gene was checked and validated. Due to the putative role of JsWRKY51 in regulating the biosynthesis of β-ocimene in *J. sambac*, the main compound (trans-β-ocimene) was measured. Furthermore, the *35S::JsWRKY51-GFP* constructs and empty vector (control) were also introduced into *J. sambac* stem-induced calli using the *Agrobacterium*-mediated transient transformation method, following the procedure developed by Chen et al., 2023 [7]. Briefly, about 3–4 days after transformation, the expression of the *JsWRKY51* gene in *J. sambac* calli (30 days of cultivation on growth medium) was checked and validated. 

After successful transformation, the transgenic and control calli (empty vector) were used for the measurement of trans-β-ocimene content. All measurements (with three biological repeats) were performed using the gas chromatography–mass spectrometry (GC-MS) platform by Guocangjian Biotechnology Co., Ltd. (Tai’an, Shandong, China). Briefly, the 100 mg powder of *JsWRKY51*-transgenic tobacco leaves, *J. sambac* calli, and controls were separately transferred into a 20 mL headspace vial containing 10 μL of an internal standard and 2 mL of a saturated NaCl solution. Each vial was heated at 150 °C for 30 min, and the SPME Arrow was then exposed to the headspace of the samples for 10 min for a gas chromatography equipped with mass spectrometer (GC-MS) analysis. The sample was injected into the capillary column (DB-624 MS, 30 m × 0.25 mm × 1.4 μm) with an auto sampler (AOC 6000). A Shimadzu GC 2010 Plus equipped with GCMS-TQ8040 was used for identification and quantification. The GC program was carried out under the following conditions: the initial temperature was 80 °C for 1 min, then increased to 250 °C at a rate of 9 °C/min, and the flow rate of helium (carrier gas) was set as constant (1.64 mL per min). Mass spectra were obtained in the full scan mode (mass range *m*/*z* 33–550) under auto-tuning conditions. The source parameters were as follows: ion source temperature, 200 °C; and MS transfer line, 250 °C. The data were calculated from the chromatogram peak area with the peak integration method using an MS detector. Three independent biological replicates were included for each experiment. Student’s *t*-test was used to analyze the significant difference in trans-β-ocimene content between the transgenic tobacco leaves, or calli, and controls. 

## 3. Results

### 3.1. Identification of JsWRKY Genes in J. sambac

A total of 75 putative *WRKY* genes were identified in the reference genome of *J. sambac* (cultivar, ‘double-petal’) by performing the HMM analysis. After removing the protein sequences with incomplete WRKY domains (based on pfam and CDD), we found 72 *WRKY* genes with complete WRKY domains in the *J. sambac* genome and named them according to their chromosomal locations. Among 72 WRKY proteins, JsWRKY10 was found to have the fewest amino acids with 77 aa, while JsWRKY38 had the most (732 aa). The proteins had MWs ranging from 8.91 (JsWRKY10) to 81.08 kDa (JsWRKY38), and their pI values ranged from 4.73 (JsWRKY64) to 10.39 (JsWRKY10). All JsWRKYs were predicted to localize in the nuclear region. Further gene characteristics including gene names, gene IDs, chromosome locations, lengths of protein sequences, isoelectric points (pI), and molecular weights (MWs) are listed in Table S1. In addition, we found that a majority of *JsWRKY* genes (58/72) identified in our study have best hits with the *JsWRKY* genes identified in single-petal *J. sambac* [11] (Table S2). 

### 3.2. Multiple Sequence Alignment, Phylogenetic Analysis, and Classification of JsWRKY Genes

Multiple sequence alignment was used to examine the conserved WRKY domains (about 60 aa) of 72 JsWRKY proteins (Figure S1). Firstly, we compared the WRKY domain of seven representative *Arabidopsis* proteins (AtWRKY58, 40, 61, 50, 74, 85, and 54) from each of the groups or subgroups. The results demonstrate that the sequences in the WRKY domain of 69 JsWRKY proteins are highly conserved WRKYGQK sequences, while the others (JsWRKY11, JsWRKY39, and JsWRKY54) vary by a single amino acid (Figure S1).

**Figure 1:**
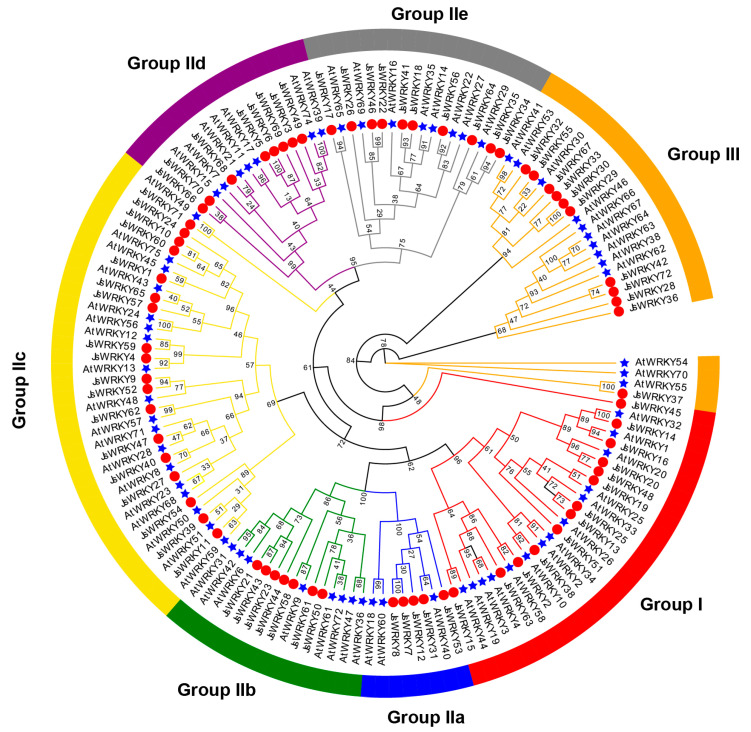
Phylogenetic analysis of WRKY proteins from *J. sambac* and *A. thaliana*. The unrooted phylogenetic tree was constructed using IQ-TREE (version 1.6.9) with the maximum likelihood method with bootstrap values of 1000. Blue stars and red circles indicate the AtWRKY proteins and JsWRKY proteins, respectively. The different-colored arcs indicate different groups (or subgroups) of WRKY proteins.

The phylogenetic analysis showed that jasmine WRKY domains could be categorized into three large groups (I, 13 members; II, 48 members; III, 11 members), and all are well clustered with the defined WRKY groups in *Arabidopsis* (Figure 1). Ten members of group I all contained two WRKY domains (WRKYGQK) and C_2_H_2_-type zinc finger motifs, whereas the other three members (JsWRKY13, JsWRKY19, and JsWRKY45) contained only one WRKY domain and C_2_H_2_-type zinc finger motifs. Group II can be further clustered into five subgroups: 5 WRKY proteins belong to IIa, 7 to IIb, 18 to IIc, 8 to IId, and 10 to IIe. Almost all members (except JsWRKY10, JsWRKY54, and JsWRKY24 in group IIc, and JsWRKY58 in group IIb) in group II contained one WRKY domain and a C_2_H_2_-type zinc finger motif. Moreover, all 11 members of group III contained a single WRKY domain and a C_2_HC-type zinc finger motif. 

### 3.3. Gene Structure and Conserved Motifs of Jasmine WRKY Gene Family

**Figure 2:**
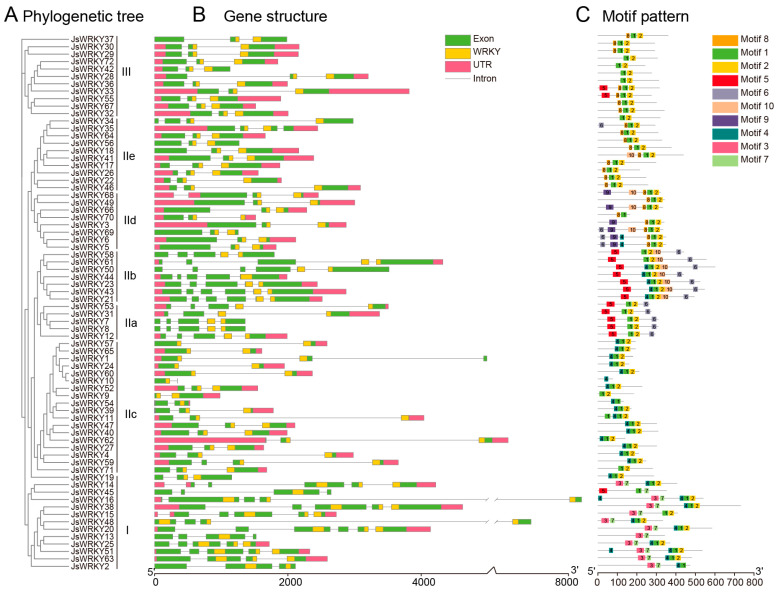
Phylogenetic tree, gene structure, and architecture of conserved protein motifs in *WRKY* genes from *J. sambac*. (**A**) The phylogenetic tree of WRKY protein sequences was constructed using IQ-TREE. (**B**) Gene structures of *WRKY* genes. The green box indicates the exon; gray line indicates the intron; and pink box indicates the 5′- and 3′-regions (UTR). The WRKY domain is highlighted with an orange box. (**C**) Distribution of conserved motifs in WRKY proteins. The motifs (named 1–10) are shown in different colored boxes.

The exon–intron organizations of all the identified *JsWRKY* genes were characterized by their phylogenetic relationships in jasmine (Figure 2A). All *JsWRKY* genes contained two to six exons, out of which six *JsWRKY* genes (*JsWRKY14*/*15*/*20*/*21*/*43*/*50*) had the highest number of exons (six) (Figure 2B). Genes within the same group (adjacent branches of the phylogenetic tree) shared similar exon–intron structures; for instance, all members in group III had three exons and two introns. Moreover, more than 91% of *JsWRKY* genes (66/72) contained an intron in their respective WRKY domains. A MEME motif analysis was conducted to identify the motif present in JsWRKY members. A total of 10 best-conserved motifs were used for a further analysis, out of which motifs 1 (WRKY domain) and 2 (zinc finger domain) were widely distributed in almost all 72 JsWRKY proteins (Figure 2C). In general, JsWRKY members within the same groups share a similar motif composition. For example, motif 9 is specific to group IId, and motifs 3 and 7 are unique to group I (Figure 2C). Overall, the fact that JsWRKY proteins in the same subgroup had similar motif compositions suggested that the structure of the proteins in each subfamily was conserved.

### 3.4. Chromosomal Distribution, Gene Duplication, and Synteny Analysis of JsWRKY Genes

**Figure 3:**
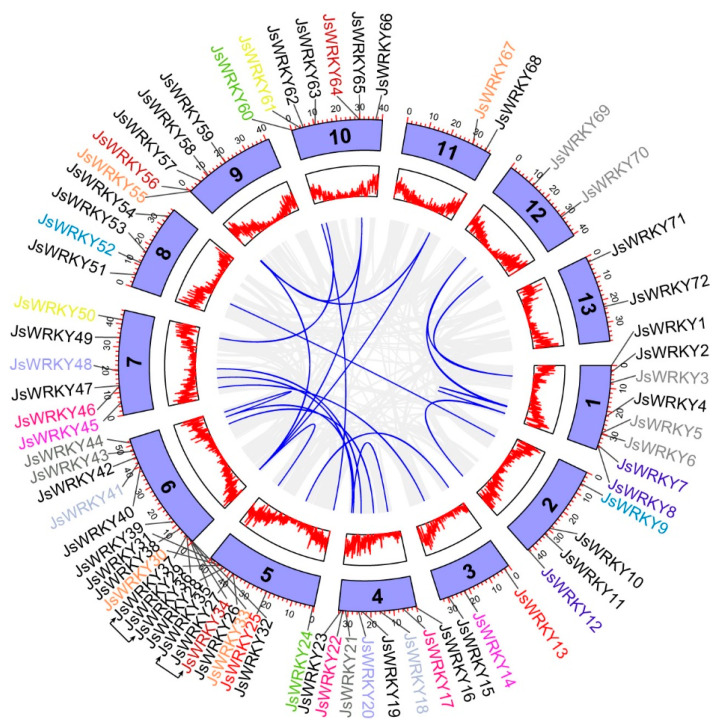
Circos plot showing chromosomal distribution and gene duplication of *WRKY* genes in *J. sambac*. Circos plot from outer to inner represents the *WRKY* genes, chromosomes, gene density, and syntenic blocks. Blue lines show the homologous gene pairs of *JsWRKY* genes on different chromosomes. Grey lines indicate all the homologous pairs between each chromosome. The tandem duplicates are linked with arrows.

The chromosomal location analysis showed that the 72 *JsWRKY* genes were distributed on 13 jasmine chromosomes (Figure 3). Chromosome 6 contained the largest number of *JsWRKY*s (13), whereas chromosomes 11, 12, and 13 each had only 2 genes. However, there is no evidence of positive correlations being observed between chromosome length and the number of *JsWRKY* genes. Based on the defined tandem duplication event [28], *JsWRKY36/37* and *JsWRKY26/27* were clustered into two tandem duplication event regions on jasmine chromosomes 5 and 6, respectively. In particular, 25 segmental duplication events with 35 *JsWRKY* genes were also identified, showing the extensive segmental duplication events in the jasmine genome. In addition, the Ka/Ks ratios of *JsWRKY* gene pairs were calculated (Table S3). All segmental and tandem duplicated *JsWRKY* gene pairs had Ka/Ks < 1, suggesting the jasmine *WRKY* gene family might have experienced strong purifying selective pressure during evolution.

### 3.5. Expression Profiling of JsWRKY Genes during Flower Scent Release 

**Figure 4:**
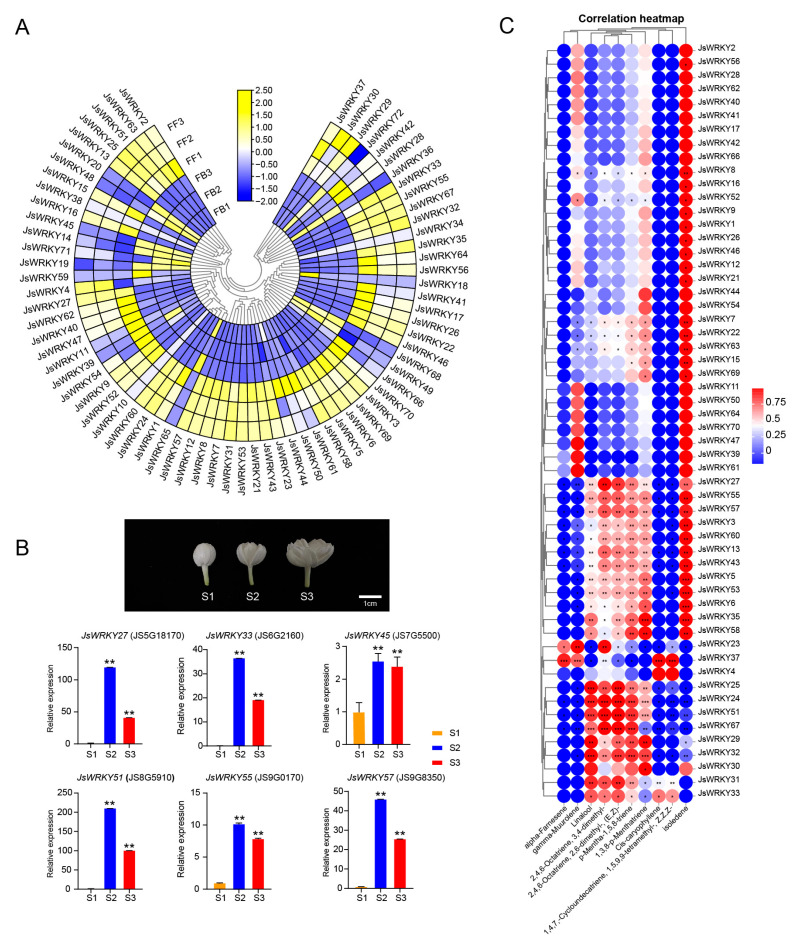
Expression patterns of *JsWRKY* genes from buds to full-blooming stages. (**A**) Heat-map representation of gene expression of *JsWRKY*s from the RNA-seq data [7] between the flower buds (FB) and full-blooming flowers (FF). *JsWRKY* gene expression is shown in three biological replicates. (**B**) *JsWRKY* gene expression validation with qRT-PCR in different blooming stages (S1 to S3). *JsWRKY27* and *JsWRKY51* exhibited dramatic changes in their expression (more than 100-fold changes in S2) among the three flower stages. The data are presented as means, with error bars indicating SE. The asterisk indicates a significant difference according to one-way ANOVA followed by Tukey’s multiple comparison test. ** *p* < 0.01. (**C**) Correlation analysis of *JsWRKY* expression and emission of 10 terpenoid compounds (sesquiterpenes and monoterpenes, data obtained from [7]). All terpenoid compounds were significantly increased in full-blooming flowers compared to flower buds. *, **, and *** indicate the significance of correlation between the terpenoid compound and *JsWRKY* expression at the 0.05, 0.01 and 0.001 levels, respectively.

The expression patterns of *JsWRKY* genes in flowers at the bud stage and full-blooming stage were examined with the RNA-seq data retrieved from the jasmine genome [7]. Among the 72 *JsWRKY*s, 77.8% (56/72) of *JsWRKY*s exhibited the highest expression in the full-blooming flower (Figure 4A; Table S4, data obtained from Chen et al., 2023 [7]), at which the volatile aroma compounds were also abundant based on our previous results (Table S5, data obtained from Chen et al., 2023 [7]). For example, *JsWRKY5*, *13*, *21*, *33*, *51*, and *57* genes are expressed at low levels in buds but high levels in full-blooming flowers. Further qRT-PCR experiments showed that *JsWRKY27* and *JsWRKY51* were dramatically upregulated at the semi-blooming (S2) and full-blooming (S3) flowering stages (Figure 4B). Terpenoids are the largest class of floral volatiles; therefore, we further focused on the significant changes in terpene components, including the monoterpene (linalool) and sesquiterpene (α-farnesene and γ-muurolene), during the transition stage of buds to full-blooming flowers, which were retrieved from previous metabolomics data (Table S5, data obtained from Chen et al., 2023 [7]). Through the gene–metabolite correlation analysis, we observed a significant correlation between the expression patterns of *JsWRKY*s and changes in volatile components (*p* < 0.05) (Figure 4C). For example, the expression patterns of *JsWRKY25*, *JsWRKY24*, *JsWRKY51*, *JsWRKY67*, *JsWRKY29*, *JsWRKY31*, and *JsWRKY32* were positively correlated with the change in linalool content. In contrast, *JsWRKY24*, *JsWRKY51*, and *JsWRKY67* were negatively correlated with cis-caryophyllene. 

### 3.6. Correlation Analysis of JsWRKY and JsTPS Expression Patterns and Cis-Element Analysis of JsTPSs

**Figure 5:**
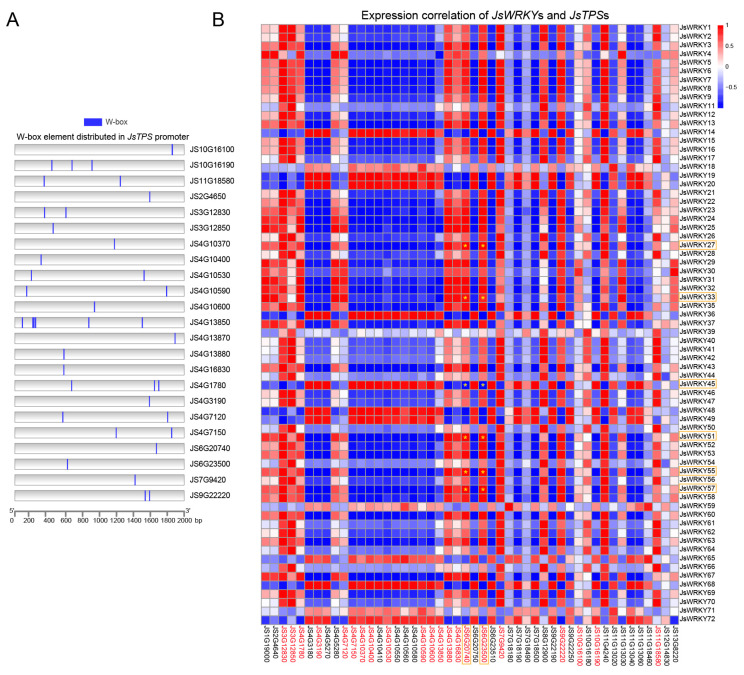
Prediction of the W-box *cis*-element and correlation analysis. (**A**) Prediction of the W-box cis-element in the *JsTPS* promoter distribution. The blue box indicates the W-box element presented in the promoter region (2000 bp) of *JsTPS* genes in *J. sambac*. (**B**) Correlation analysis between the expression of the *JsWRKY* and *JsTPS* genes. A red box denotes positive correlation, and a blue box denotes negative correlation. The yellow asterisk (*) within the box indicates the significant correlation (*p* < 0.01 and Pearson correlation coefficient > 0.95) between the *JsWRKY* and *JsTPS* genes. The IDs of *JsTPS* genes containing W-box cis-elements in the heat map are highlighted in red letters. The key genes that were validated with qRT-PCR are marked with the orange rectangular box.

Terpene synthase (TPS), the essential enzyme for terpene biosynthesis, plays a crucial role in the production of floral volatiles. In the jasmine genome, our previous study identified a total of 47 *TPS* genes with two conserved domains and their expression patterns between FBs and FFs (Table S6, data obtained from Chen et al., 2023 [7]). In this study, to screen the *JsTPS* genes that are potential targets for regulation by JsWRKYs, we first performed a *cis*-element analysis of the *JsTPS* promoters and found that the promoter regions of 49% (23/47) of the *JsTPS* genes contained one or more W-box elements (Figure 5A), showing their potential as DNA binding sites for JsWRKYs. We further performed a correlation analysis of the expression patterns of all *JsTPS* and *JsWRKY* genes. In general, most *JsTPS* genes exhibited a negative (blue) correlation with the expression of most *JsWRKY* genes. However, more than 60% (14/23) *JsTPS* genes containing W-box elements were strongly positively correlated with *JsWRKY*s in terms of their expression patterns (Figure 5B). For example, the expression patterns of *JS6G202740* and *JS6G23500* were significantly positively correlated with changes in *JsWRKY27*, *JsWRKY33*, *JsWRKY51*, *JsWRKY55*, and *JsWRKY57*, whereas *JS4G3190* and *JS4G7150* were negatively correlated with these *JsWRKY* genes. 

Moreover, our previous qRT-PCR results indicated that five *JsTPS* genes (*JS6G23500*, *JS6G23510*, *JS5G5280*, *JS4G5270*, and *JS6G20740*) have significantly higher expression in full-blooming flowers than in flower buds [7]. Accordingly, the five *JsTPS* genes were focused on for a further analysis in this study. Interestingly, *JS6G20740* (*JsTPS3*) encoding β-ocimene synthase contained the W-box element and exhibited the highest expression levels in full-blooming flowers. Notably, the expression pattern of the *JsTPS3* gene was highly consistent with that of *JsWRKY51*, which increased by more than 100-fold in full-blooming flowers, as indicated with RNA-seq (Table S3, data obtained from Chen et al., 2023 [7]) and qRT-PCR data (Figure 4B). 

### 3.7. Subcellular Localization of JsWRKY51

**Figure 6:**
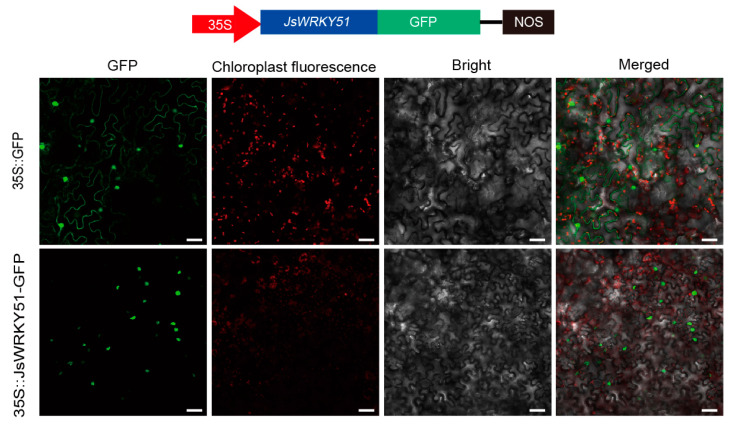
Subcellular localization of JsWRKY51 in tobacco leaves. Bar represents 50 μm.

Given the potential function of JsWRKY51 in transcription regulation of *JsTPS*s, we constructed *p35S::JsWRKY51-GFP* and then used the tobacco leaf to examine their subcellular localization (Figure 6). The GFP fluorescence of both *p35S::GFP* and *p35S::JsWRKY51-GFP* was observed only in the nucleus, demonstrating that the JsWRKY51 transcription factor was localized in the nucleus.

### 3.8. Overexpression of JsWRKY51 Promotes β-Ocimene Accumulation in Tobacco Leaves and in Jasmine Calli

**Figure 7:**
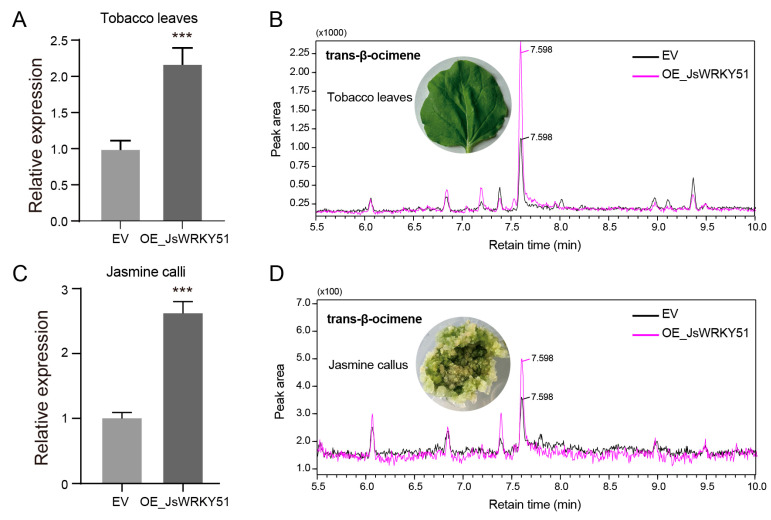
Overexpression of *JsWRKY51* enhances β-ocimene accumulation in tobacco leaves and in *J. sambac* calli. (**A**) qRT-PCR validation of *JsWRKY51* expression in transgenic tobacco leaves. (**B**) GC-MS peaks of trans-β-ocimene in *JsWRKY51*-transgenic tobacco leaves. (**C**) qRT-PCR validation of *JsWRKY51* expression in transgenic jasmine calli. (**D**) GC-MS peaks of trans-β-ocimene in *JsWRKY51*-transgenic jasmine calli. EV, empty vector; OE, overexpression. Data (**A**,**C**) are the mean values (*n* = 3), and error bars represent the standard error (SE). The asterisk (***) above the error bar indicates the significant differences between EV and OE_*JsWRKY51* at the 0.001 level using the Student *t*-test.

We constructed the recombinant plasmid carrying 35S::*JsWRKY51* and performed transient overexpression in tobacco leaves. *JsWRKY51* exhibited a significantly higher expression in the overexpression tobacco leaves (OE-*JsWRKY51*) than in empty vector-transformed (EV) leaves, indicating successful transformation (Figure 7A). Through GC-MS detection, the content of trans-β-ocimene in OE-*JsWRKY51* tobacco leaves was increased by 34.78% compared to the EV (Figure 7A). Furthermore, we also successfully transiently transformed the *35S::JsWRKY51* plasmid into jasmine calli, which resulted in a significant accumulation of the trans-β-ocimene content (by approximately 25.78%) in jasmine OE-*JsWRKY51* calli (Figure 7C,D). We also examined the expression level of *JsTPS3* and observed a significant increase in expression in transgenic jasmine calli (Figure S3). These results suggest that JsWRKY51 plays a crucial role in β-ocimene biosynthesis, possibly by regulating the expression of *JsTPS3*. 

## 4. Discussion

The WRKY TFs comprise a large gene family that is ubiquitous in all plant species. As more genomes have been sequenced, genome-wide identification analyses of WRKY TFs have been conducted in many species [29]. The regulatory functions of WRKY TFs in plants have been well recognized, including defense against abiotic and biotic stresses, growth and development, and secondary metabolism. *J. sambac* is well known around the world as a fragrant plant with sweet-scented flowers, of which terpenes are the most important volatile components [7]. WRKYs significantly participate in regulating the terpene biosynthesis pathway in plants [30,31]. Xu et al. (2021) [6] identified three *WRKY* genes potentially associated with abiotic and biotic stress in the *J. sambac* genome (cultivar, Trifoliatum). Recently, the genome-wide identification of 69 *WRKY* genes has been reported in the single-petal phenotype of *J. sambac* (cultivar, ‘Danbanmoli’). However, genome-wide analyses of *WRKY* genes and their regulation of terpenoids have not been reported for the double-petal *J. sambac* (cultivar, ‘double petal’). Our study characterizes genome-wide WRKY TFs in double-petal *J. sambac* and reveals their significance in regulating terpene biosynthesis. 

The variation in the number of gene family members is a crucial mechanism that shapes adaptive natural variation during species evolution [32]. The number of *WRKY* genes varies among different species, ranging from 1 or a few genes in green algae to over 30 genes in the earliest land plant mosses and over 100 genes in some flowering plants [15]. In the present study, we discovered 72 *WRKY* genes in the double-petal *J. sambac* genome, the number of which was slightly higher than that of *JsWRKY* genes (69 *JsWRKY*s) in the single-petal *J. sambac* genome [11]. A further comparative analysis of protein sequences suggested that a majority of *JsWRKY*s (>80%) identified in this study share extremely high homology with *JsWRKY*s obtained from single-petal *J. sambac* [11]. In contrast, the *JsWRKY* genes exhibiting low similarities in sequences between single-petal and double-petal *J. sambac* may indicate their different biological functions. In addition, the number of *JsWRKY*s (72) identified in our study is significantly higher than the number of *WRKY* genes in basal eudicots *Vitis vinifera* (59) [33]; however, it is significantly lower than those in the Oleaceae family *O. fragrans* (154) [16]. 

Tandem and segmental duplication events were also the main contributors to the expansion of the *WRKY* gene family [34]. For example, the amplification of group III *WRKY* genes in *Oryza sativa* ssp. *japonica* and *Oryza nivara* arose from segmental and tandem gene duplication [35,36]. Nearly half of the *TaWRKY* genes emerged from segmental duplication in wheat [37]. In this study, we found 49% (35/72) *JsWRKY*s from all across the groups (I–III) exhibited gene amplification through segmental duplication, whereas only 4 *JsWRKY*s were tandemly duplicated. Notably, most of these *JsWRKY* gene pairs have *Ks* values ranging from 1.0 to 1.5, which coincides with the timing of WGD (*Ks* = 1.1–1.3, 46.2–55.0 million years ago) in *Jasminum* [7]; therefore, the *Jasminum* WGD event may have contributed to the occurrence of *JsWRKY* segmental duplicates. Collectively, the WGD and segmental duplication events facilitated the expansion of WRKY family genes in *J. sambac*. Additionally, we speculate that the *JsWRKY* gene family may have been subjected to significant purifying selection forces throughout evolution because almost all *JsWRKY* orthologous gene pairs had Ka/Ks < 1. 

Dicotyledons have experienced less evolutionary loss of the WRKY conserved domain than monocotyledons [38,39]. For example, rice has been reported to have nine variants [40], and the common bean contains WRKYGKK, WRKYGEK, WKKYEDK, and WKKYCEDK [41]. However, the WRKY domains of *J. sambac* only contained two variants, the conserved heptapeptide WRKYGQK and its variant WRKYGKK, which further validated this occurrence. Furthermore, the two WRKY variants constitute two separate clades (WRKYGKK and WRKYGQK) of the group IIc, which is consistent with the eight WRKYGKK variants belonging to group IIc observed in *O. fragrans* [16], indicating that these *JsWRKY* genes with different variants may possess different biological functions. Previous studies have demonstrated that the WRKY TFs regulate target genes through binding W-box elements, whereas changes in the WRKYGQK motif may affect DNA-binding interactions with downstream genes [14,42]. As a result, the exploration of the functional and binding properties of these three *JsWRKY*s might be worthy for further investigation. Additionally, group I WRKY proteins usually contain two WRKY domains [43], of which the C-terminal WRKY domain functions in DNA binding, but the function of the N-terminal WRKY domain remains unclear [44]. Our study found that JsWRKY13 and JsWRKY45 proteins within group I possess only one domain in their N-terminal, indicating their potential functional divergence among the members within group I. 

The presence of exon–intron gene structures provides evidence of the historical evolution of gene families and serves as the foundation for categorizing them phylogenetically [45]. A similar exon–intron distribution pattern of family genes within one subgroup indicates functional similarity among members [46]. The present study showed that *JsWRKY*s from group I contain more introns than other groups, which implies that it is more likely that other groups evolved from group I. This conclusion was also supported with the hypothesis that all *WRKY* genes originated from group I C-terminal WRKY domains based on the origin analysis of subfamilies phylogenetically [14,47]. 

The main volatile aromatic compounds of *J. sambac* flowers are composed of terpenes, phenylpropanes, and fatty acid derivatives, of which terpene compounds (sesquiterpenes, monoterpenes, and isopentane) are the most important volatile substances [7]. The aromatic substances in jasmine flowers determine their quality and economic value for industrial applications. During the flower stage of *J. sambac*, a large number of volatile aromatic compounds are gradually synthesized and accumulated from flowers at the bud-to-full-blooming stages [7,9]. Studies have revealed that the expression patterns of WRKY TFs were correlated with the change in the buildup of aromatic compounds. For instance, the abundance of *OfWRKY7*/*19*/*36*/*84*/*139* was most closely associated with the trends in aroma changes in *O. fragrans* [16]. Similarly, the expression pattern of *OfWRKY19* was positively correlated with the accumulation of β-ocimene and (E,Z)-2,6-dimethylocta-2,4,6-triene, whereas *OfWRKY7* was negatively correlated with ocimene and its derivatives [16]. In this study, we observed that the majority of *JsWRKY* genes (e.g., *JsWRKY21*, *JsWRKY27*, *JsWRKY51*, *JsWRKY55*, and *JsWRKY57*) expressed abundantly in full-blooming flowers, which was significantly associated with the accumulation of multiple terpenoid compounds (monoterpenes and sesquiterpenes) at the blooming stage. In particular, qRT-PCR showed that the fold change in *JsWRKY27* and *JsWRKY51* expression was the highest in semi-blooming and full-blooming flowers, suggesting their possible involvement in the regulation of terpene volatile accumulation in *J. sambac*. 

WRKY TFs are essential regulators of secondary metabolism, including phenylpropanoid, terpene, and alkaloid metabolism [18]. They can activate or inhibit transcription of secondary metabolic processes by recognizing and binding the W-box (TTGACT/C) in their target genes [31]. For instance, *Artemisia annua* WRKY1 (AaWRKY1) can promote the transcription of *ADS*, *CYP71AV1,* and *DBR2* [12,48], and glandular trichome-specific WRKY1 (AaGSW1) directly binds to W-boxes in the promoters of *CYP71AV1* and *ORA*, both of which positively promote artemisinin (a type of sesquiterpene lactone) biosynthesis [31,49]. Moreover, the TPS family genes, being involved in MVA and MEP pathways, are responsible for the synthesis of various terpene volatiles [50,51]. In tomatoes, SlWRKY73 transactivates the promoters of *SlTPS3*, *SlTPS5*, and *SlTPS7* monoterpene synthase genes [52], thus potentially regulating monoterpene synthesis. In *J. sambac*, genome-wide *TPS* genes have been identified, and *JsTPS3* (JS6G20740) was demonstrated to be involved in the biosynthesis of β-ocimene, as an important component of the *J. sambac* floral scent [7]. Based on the identified jasmine *TPS* genes from Chen et al. (2023) [7], a significantly negative correlation between most *JsTPS* and *JsWRKY* genes in expression patterns was observed. In contrast, the present study also showed that most *TPS* genes (14/23) containing W-box elements were strongly positively correlated with some *JsWRKY*s (*JsWRKY27*, *JsWRKY33*, *JsWRKY51*, *JsWRKY55*, and *JsWRKY57*) in terms of their expression patterns. This result suggested that these JsWRKYs may be involved in regulating *JsTPS*s for the biosynthesis of different terpene volatiles. Overexpression of nucleus-localized *JsWRKY51* both in tobacco leaves and jasmine calli significantly enhanced the accumulation of β-ocimene, demonstrating that *JsWRKY51* plays a role in the biosynthesis of β-ocimene. Consistently, β-ocimene synthesis-related *JsTPS3* containing the W-box *cis*-element also exhibited upregulated expression in transgenic jasmine calli, indicating that *JsTPS3* may be regulated by JsWRKY51. Nevertheless, understanding the mechanism by which JsWRKY51 enhances the synthesis of β-ocimene, probably by binding specific *TPS* promoters, still needs further investigation.

## 5. Conclusions

A total of 72 WRKY family genes were identified in the *J. sambac* genome. The chromosomal locations, classification, and gene structure were systematically characterized. The synteny analysis revealed that extensive segmental duplication is the main driving force for the expansion of *JsWRKY* genes. A majority of *JsWRKY*s exhibited higher expression in flowers at the bud stage compared to that at full-blooming stages. The correlation and qRT-PCR analysis indicated the involvement of *JsWRKY*s in aroma synthesis by regulating terpene volatile compounds because the expression patterns of *JsWRKY*s were correlated with the emission patterns of terpene compounds and the expression of *JsTPS*s. Overexpression of *JsWRKY51* in tobacco leaves and jasmine calli promotes the accumulation of β-ocimene, highlighting the role of *JsWRKY51* in the regulation of β-ocimene biosynthesis. Furthermore, the expression of β-ocimene-synthesis-related *TPS3* (with the presence of W-box cis-element) significantly increased in *JsWRKY51* transgenic calli, indicating a potential regulatory module of JsWRKY51-*TPS3* contributing to the biosynthesis of volatile β-ocimene compounds. Further studies are needed to reveal the regulatory mechanism of *JsWRKY*s in aroma synthesis in *Jasminum*.

## Data Availability

The data presented in this study are available in this article (and supplementary material).

## Supplementary Materials

The following supporting information can be downloaded at: https://www.mdpi.com/article/10.3390/biom13121679/s1, Figure S1: Multiple sequence alignment of JsWRKY with selected AtWRKY domain amino acid sequences. It shows a conserved WRKY domain and zinc-finger structure. Figure S2: Cloning and validation of the full-length CDS of *JsWRKY51*. (A) PCR amplification. (B) Sequence validation. Figure S3 Expression analysis of *JsTPS3* responsible for β-ocimene biosynthesis in transgenic jasmine calli. The data are the mean values, and error bars represent the standard error (SE). The asterisk (**) above the error bar indicates the significant differences between EV and OE_*JsWRKY51* at the 0.01 level using the student *t-*test. title; Table S1: List of the identified 72 *JsWRKY* genes. Table S2: Reciprocal blast of JsWRKY protein sequences identified from single-petal (Qi et al. 2022 [11]) and double-petal *J. sambac* (this study). Table S3: Segmental and tandemly duplicated *JsWRKY* gene pairs with *Ka*/*Ks* value. Table S4: RNA-seq data of 72 *JsWRKY* genes (data obtained from Chen et al. 2023 [7]). Table S5: List of terpenoid compounds significantly increased during buds to full-blooming flowers in *J. sambac* (data obtained from Chen et al.2023 [7]). Table S6: RNA-seq data of 47 *JsTPS* genes (data obtained from Chen et al., 2023 [7]). Table S7: List of the primers used in this study.
