# Supplementary figures and images for: WRKY Transcription Factors in Jasminum sambac: An Insight into the Regulation of Aroma Synthesis
Source: Biomolecules. 2023 Nov 21;13(12):1679. doi: 10.3390/biom13121679 (PMC10742223; doi:10.3390/biom13121679)

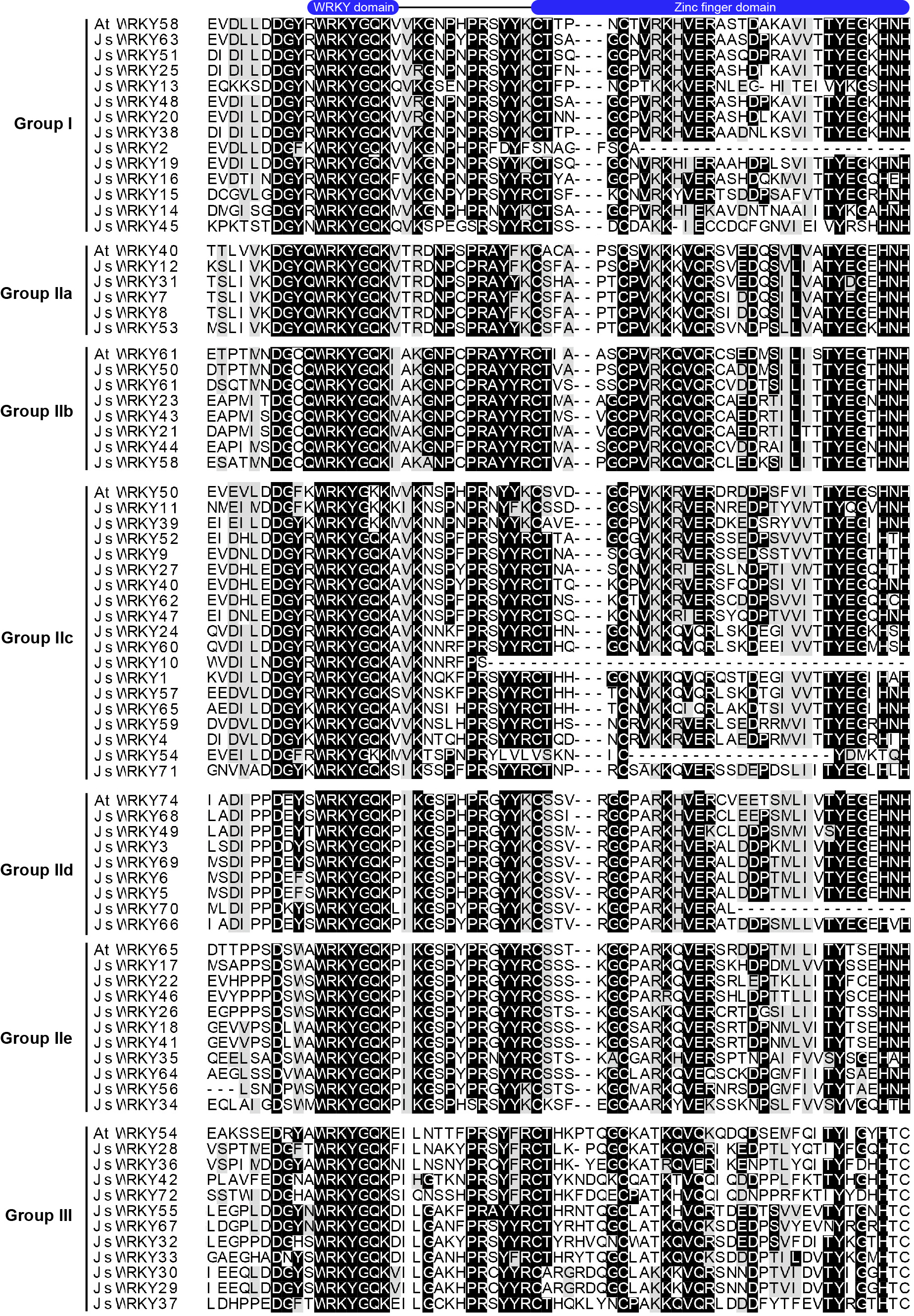

Supplement: Supplementary file 1 [file biomolecules-13-01679-s001.zip › Figure S1.jpg]

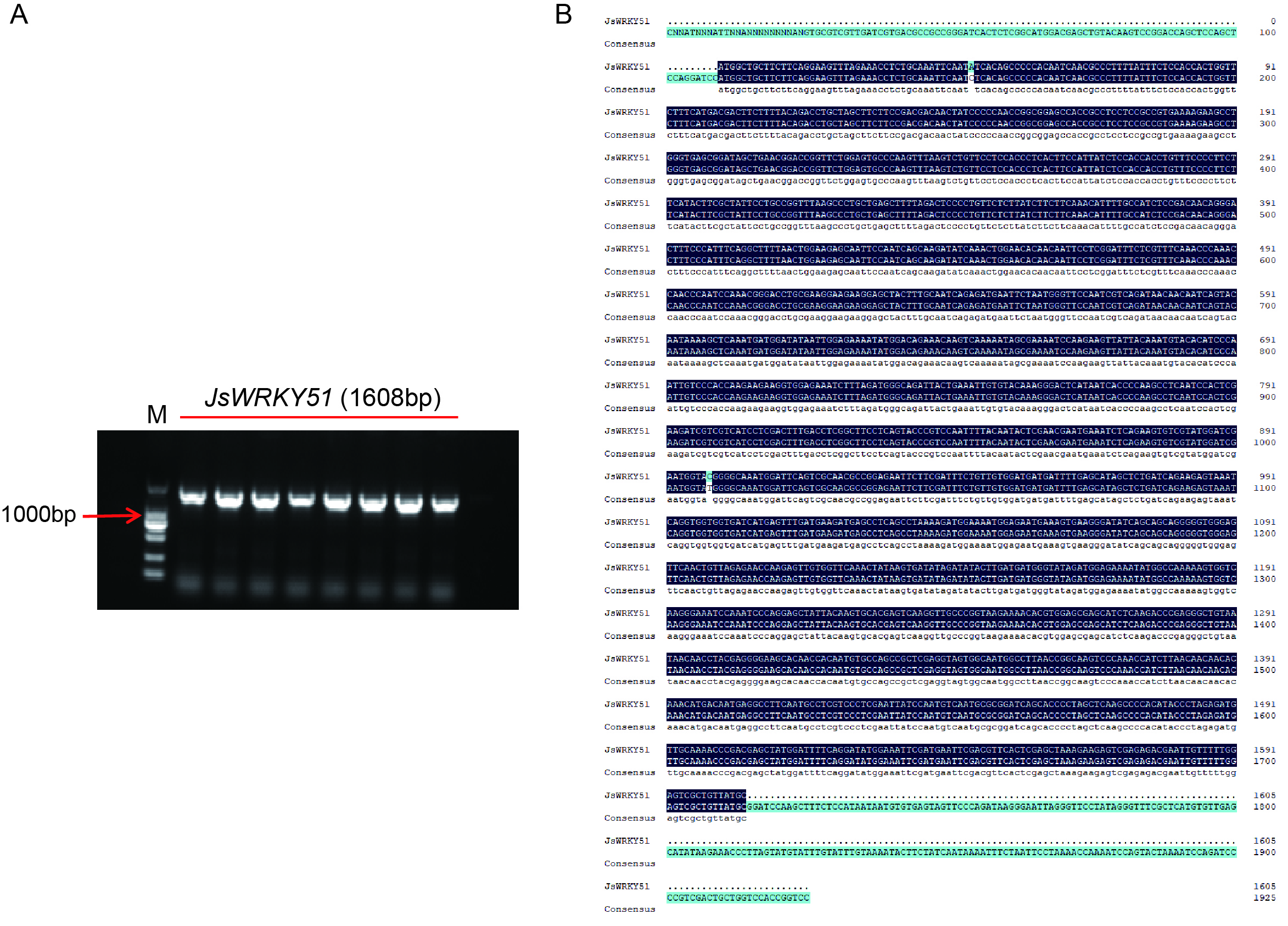

Supplement: Supplementary file 1 [file biomolecules-13-01679-s001.zip › Figure S2.jpg]

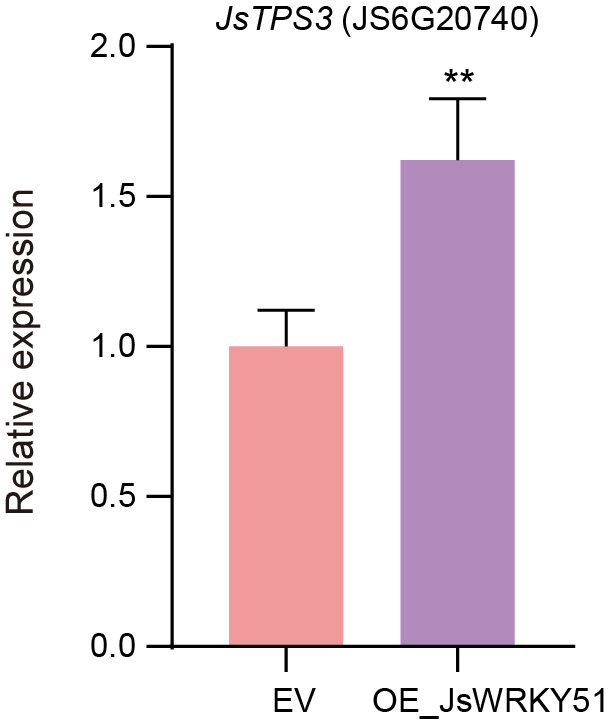

Supplement: Supplementary file 1 [file biomolecules-13-01679-s001.zip › Figure S3.jpg]
